# Locally advanced rectal cancer with dMMR/MSI-H may be excused from surgery after neoadjuvant anti-PD-1 monotherapy: a multiple-center, cohort study

**DOI:** 10.3389/fimmu.2023.1182299

**Published:** 2023-06-27

**Authors:** Renfang Yang, Tao Wu, Jiehai Yu, Xinyi Cai, Guoyu Li, Xiangshu Li, Weixin Huang, Ya Zhang, Yuqin Wang, Xudong Yang, Yongping Ren, Ruixi Hu, Qing Feng, Peirong Ding, Xuan Zhang, Yunfeng Li

**Affiliations:** ^1^ Department of Colorectal Surgery, Yunnan Cancer Hospital, The Third Affiliated Hospital of Kunming Medical University, Kunming, China; ^2^ Department of Colorectal Surgery, Sun Yat-sen University Cancer Center, State Key Laboratory of Oncology in South China, Collaborative Innovation Center for Cancer Medicine, Guangzhou, China; ^3^ Department of Gastrointestinal Surgery, The First Affiliated Hospital of Chongqing Medical University, Chongqing, China; ^4^ Department of Gastrointestinal Surgery, Honghe Prefecture Third People’s Hospital, Honghe Cancer Hospital, Gejiu, China; ^5^ Department of Imaging, Yunnan Cancer Hospital, The Third Affiliated Hospital of Kunming Medical University, Kunming, China; ^6^ Department of Pathology, Yunnan Cancer Hospital, The Third Affiliated Hospital of Kunming Medical University, Kunming, China

**Keywords:** locally advanced rectal cancer, neoadjuvant immunotherapy, programmed cell death protein-1 inhibitor, mismatch repair-deficient, clinical complete response, watch-and-wait strategy

## Abstract

**Objective:**

Examine patients with locally advanced rectal cancer (LARC) with deficient mismatch repair (dMMR) or microsatellite instability-high (MSI-H) who received neoadjuvant immunotherapy (nIT), and compare the outcomes of those who chose a watch-and-wait (WW) approach after achieving clinical complete response (cCR) or near-cCR with those who underwent surgery and were confirmed as pathological complete response (pCR).

**Methods:**

LARC patients with dMMR/MSI-H who received nIT were retrospectively examined. The endpoints were 2-year overall survival (OS), 2-year disease-free survival (DFS), local recurrence (LR), and distant metastasis (DM). The efficacy of programmed cell death protein-1 (PD-1) inhibitor, immune-related adverse events (irAEs), surgery-related adverse events (srAEs), and enterostomy were also recorded.

**Results:**

Twenty patients who received a PD-1 inhibitor as initial nIT were examined. Eighteen patients (90%) achieved complete response (CR) after a median of 7 nIT cycles, including 11 with pCR after surgery (pCR group), and 7 chose a WW strategy after evaluation as cCR or near-cCR (WW group). Both groups had median follow-up times of 25.0 months. Neither group had a case of LR or DM, and the 2-year DFS and OS in each group was 100%. The two groups had similar incidences of irAEs (P=0.627). In the pCR group, however, 2 patients (18.2%) had permanent colostomy, 3 (27.3%) had temporary ileostomy, and 2 (18.2%) had srAEs.

**Conclusion:**

Neoadjuvant PD-1 blockade had high efficacy and led to a high rate of CR in LARC patients with dMMR/MSI-H. A WW strategy appears to be a safe and reliable option for these patients who achieve cCR or near-cCR after nIT.

## Introduction

Colorectal cancer (CRC) is the most common malignancy of the digestive system, and global cancer statistics for 2020 indicated it had the third-highest incidence and the second-highest mortality rate among all cancers (1). About 60% of patients with CRC have locally advanced disease upon diagnosis (2), defined as CRC stage II (clinical T3–T4, N0) or stage III (any clinical T, N1–N2). Neoadjuvant fluorouracil-based chemotherapy and radiotherapy followed by total mesorectal excision (TME), with or without postoperative chemotherapy, is the standard treatment regimen for patients with locally advanced rectal cancer (LARC) (3, 4), and this regimen enables approximately 20% of these patients to achieve a pathological complete response (pCR) (5, 6). Nevertheless, the short- and long-term toxicities from this treatment, including defecation disorders, urinary and sexual dysfunction, surgical complications, and temporary or permanent enterostomy, can seriously reduce a patient’s quality of life (7, 8). Data from a large number of studies have confirmed that adoption of a watch-and-wait (WW) strategy by patients with rectal cancer who achieved a clinical complete response (cCR) after neoadjuvant chemoradiotherapy (nCRT) prevented surgical trauma, preserved organ function, and provided a survival benefit similar to surgery (9, 10). Even for patients with near-cCR, a previous study demonstrated that more than half of them achieved organ preservation within 3 years after a WW strategy, and their local recurrence-free survival and metastasis-free survival rates were not significantly different from those who had cCR (11). However, with the increasing availability of neoadjuvant treatment options, it is uncertain whether patients with rectal cancer who achieve a cCR or near-cCR after treatment with other neoadjuvant modalities should also adopt a WW strategy.

There is evidence that CRC patients with deficient mismatch repair (dMMR) or microsatellite instability-high (MSI-H) receive little benefit from fluorouracil-based chemotherapy (12, 13). However, these patients typically have high sensitivity to immunotherapies, such as anti-program death-1 (PD-1) antibodies, and their responses are often long-lasting (14, 15). In addition, higher rates of pathological response were achieved when immune checkpoint inhibitors (ICIs) were used with the first-line or neoadjuvant application rather than at a later time (16–18). Although there is only no more than 10% of rectal cancers are classified as dMMR/MSI-H (19–21), a complete response (CR) rate more than 60% can be achieved from neoadjuvant immunotherapy (nIT), significantly higher than from nCRT (21–23). In addition, immunotherapy leads to fewer adverse effects and almost no damage to the sphincter, reproductive organs, sexual function, or bladder (21–23). These advantages of nIT suggest that LARC patients with dMMR/MSI-H who achieved a cCR or near-cCR after nIT might benefit from a WW strategy.

Current data regarding the effect of a WW strategy after nIT for CRC are very rare. As far as we know, this is the first study to compare the survival outcomes of MSI-H/dMMR LARC patients receiving nIT treatment who opted for a WW strategy after achieving a cCR or near-cCR with those who underwent surgery and confirmed as pCR. The present study is a preliminary evaluation of the safety and feasibility of the WW strategy after nIT in these patients using data from multiple centers.

## Materials and methods

### Patient selection

This study was a retrospective, multicenter, case series study. We reviewed patients with LARC (clinical stage T3–4/N0–2/M0) and dMMR/MSI-H who received a PD-1 inhibitor (no type limitations) alone as an initial neoadjuvant treatment from January 2019 to May 2020 at the Yunnan Cancer Hospital (Third Affiliated Hospital of Kunming Medical University), Sun Yat-sen University Cancer Center, First Affifiliated Hospital of Chongqing Medical University, or Honghe Cancer Hospital (Honghe Prefecture Third People’s Hospital). All eligible patients were 18 to 75 years-old, had an Eastern Cooperative Oncology Group (ECOG) performance score of 0 to 1, and received 4 or more doses of a PD-1 inhibitor. The exclusion criteria were: suspected metastatic disease; dMMR based on immunohistochemical staining (IHC), but no evidence of MSI-H based polymerase chain reaction (PCR) testing or next-generation sequencing (NGS); active autoimmune disease or history of autoimmune disease or previous receipt of systemic biological immunotherapy.

### Data collection

Standardized electronic forms were sent to physicians in each center. Complete demographic and clinicopathological information of patients were collected, including ECOG status, family and personal history of malignant tumors, serum carcinoembryonic antigen (CEA) level, clinical and pathological stage, pathological type of CRC, degree of differentiation, mismatch repair (MMR) or microsatellite status, treatment regimen, treatment response, tumor regression grade (TRG), immune-related adverse events (irAEs), surgery-related adverse events (srAEs), follow-up, and survival.

All staging was performed according to the eighth edition of the American Joint Committee on Cancer (AJCC) (24). MMR status was determined by IHC staining for mismatch repair proteins (MLH1, MSH2, MSH6, and PMS2) in biopsy tissues before treatment. Microsatellite status was determined by PCR or NGS technology. Among them, PCR was used as the “gold standard” to determine microsatellite status by analyzing the five consensus tumor microsatellite loci: two mononucleotides (*BAT25* and *BAT26*) and three dinucleotides (*D5S346*, *D2S123*, and *D17S250*), and NGS was recommended as a second-line method for microsatellite status detection (25). Studies have shown that the sensitivity and specificity of IHC for MMR or PCR for MSI were both above 90%, and the concordance between the two methods is approximately 90% (26, 27). In addition, NGS-based MSI testing results to be up to 99% concordant with conventional PCR and 92.4% concordant with double confirmed IHC staining (28).

### Treatment methods

All eligible patients started neoadjuvant anti-PD-1 monotherapy after diagnosis, and none of them were treated with combined radiotherapy, chemotherapy, targeted therapy, or an additional ICI. Each patient received 200 mg of a PD-1 inhibitor by intravenous infusion every 3 weeks until tumor regression to feasible R0 resection, cCR, or near-cCR. There were no limits on the type of PD-1 inhibitors, and these included pembrolizumab, sintilimab, and tislelizumab.

The timing and procedure of the operation, need for adjuvant immunotherapy (aIT), and treatment course were determined by the head surgeon after a comprehensive evaluation of the patient’s response to treatment and general condition. Patients who achieved a cCR or near-cCR after nIT were informed of the benefits and risks of the therapeutic alternatives, and most of them expressed a strong desire for the preservation of organ function and avoidance of enterostomy. Given the long-lasting response of immunotherapy and the inconsistency between imaging and pathological evaluation (for example, some patients with imaging evaluation of PR were pathologically confirmed as pCR in our previous study (22)), doctors agreed on their request for exemption from surgery. Before undergoing the WW strategy, they were informed that a WW strategy after cCR, especially after near-cCR following nIT is not currently a standard therapy and signed informed consent documents. All of them were vigilant about regular follow-up and adherence to the recommended surveillance, with consent for radical resection once the disease progresses.

### Treatment response and survival outcomes

Response to treatment was assessed by magnetic resonance imaging (MRI) of the pelvic region, transrectal ultrasound (TRUS), enhanced CT examination of chest and abdomen, digital rectal examination (DRE), serum carcinoembryonic antigen (CEA), endoscopy, and selective biopsy of any residual mass or scar. The standard of efficacy evaluation was based on the Response Evaluation Criteria in Solid Tumors RECIST Version 1.1 (RECIST 1.1) (29). Because there is currently no unified international diagnostic standard for cCR and near-cCR, the standards in our study were based on the Sao Paulo criteria (30), the criteria in ESMO guidelines (4), and the Memorial Sloan Kettering Regression Schema (31), with fine-tuning according to the actual situation. The specific diagnostic criteria for cCR and near-cCR are in **Supplementary Table S1**.

The indicators of pathological efficacy after nIT were ypTNM stage and TRG. According to the AJCC system, TRG-0 refers to no residual tumor cells, TRG-1 refers to a single tumor cell or a small group of tumor cells, TRG-2 refers to residual cancer with desmoplastic response, and TRG-3 refers to minimal or no evidence of tumor response (24). The pCR was defined as tumor regression induced by neoadjuvant therapy, with no viable tumor cells in the resected primary tumor sample and all sampled regional lymph nodes (pCR = TRG-0 = ypT0N0M0) (24). Major pathological response (MPR) was considered to be tumor regression with 10% or less pathological residual tumor (MPR = TRG-0 + TRG-1) (24).

The primary survival outcomes were 2-year overall survival (OS), 2-year disease-free survival (DFS), local relapse (LR), and distant metastasis (DM). For DFS, the date of the last nIT treatment in the WW group and the date of surgery in the surgery group was the start date; the date of the last follow-up or the first recurrence, metastasis, or death from any cause was the termination date. OS was measured from the date of the first nIT treatment to the date of death. LR was defined as the presence of rectal adenocarcinoma inside the pelvis at the anastomosis site, presacral area, or pelvic lymph node. DM was defined by rectal adenocarcinoma recurrence that spread to an area or organ outside the pelvis (liver, lung, ovary, distant lymph node, etc.).

Treatment-related adverse events were also recorded. Immune-related adverse events (irAEs) refer to adverse events related to immunotherapy that occurred from the beginning of nIT until 90 days after the last dose of the PD-1 inhibitor, and were graded using the Common Terminology Criteria for Adverse Events (CTCAE) version 5.0 (32). Surgery-related adverse events (srAEs) refer to complications directly or indirectly related to surgery from the day of surgery to 30 days after surgery, and were graded using the Clavien-Dindo grading evaluation standard (33).

### Follow-up methods

For patients who underwent TME, follow-up was performed according to international guidelines (3). For patients managed by the WW strategy, a more intensive follow-up protocol was used due to the lack of uniform standards. This follow-up consisted of measurements of serum CEA and DRE every 2 to 3 months during the first two years; T2-weighted and diffusion-weighted MRI of the pelvis, TRUS, and complete colonoscopy every 3 months during the first year, and every 6 months during the subsequent 4 years; and enhanced CT examination of chest and abdomen every 6 months. Biopsy was performed selectively to examine residual nodularity or scarring from the colonoscopy examination, and abnormalities were identified on the cross-sectional imaging. The duration of follow-up was calculated as the time from the last day of the last treatment (last nIT, or aIT, or surgery) until the event of interest or the last follow-up date.

### Statistical analysis

Continuous numerical variables were presented as medians and ranges, and compared using an independent samples *t*-test. Categorical variables were presented as numbers and percentages, and compared using the Chi-square test or Fisher’s exact test. Cumulative DFS, OS, LR, DM were presented using Kaplan-Meier curves, and the WW and pCR groups were compared using the Wilcoxon test. All statistical tests were two-sided, and results were considered statistically significant when the *P* value was less than 0.05. Statistical analyses were performed using the Statistical Package for the Social Sciences Program (SPSS Inc. Chicago, IL, version 26.0 for Mac).

## Results

### Characteristics of patients

**Figure 1:**
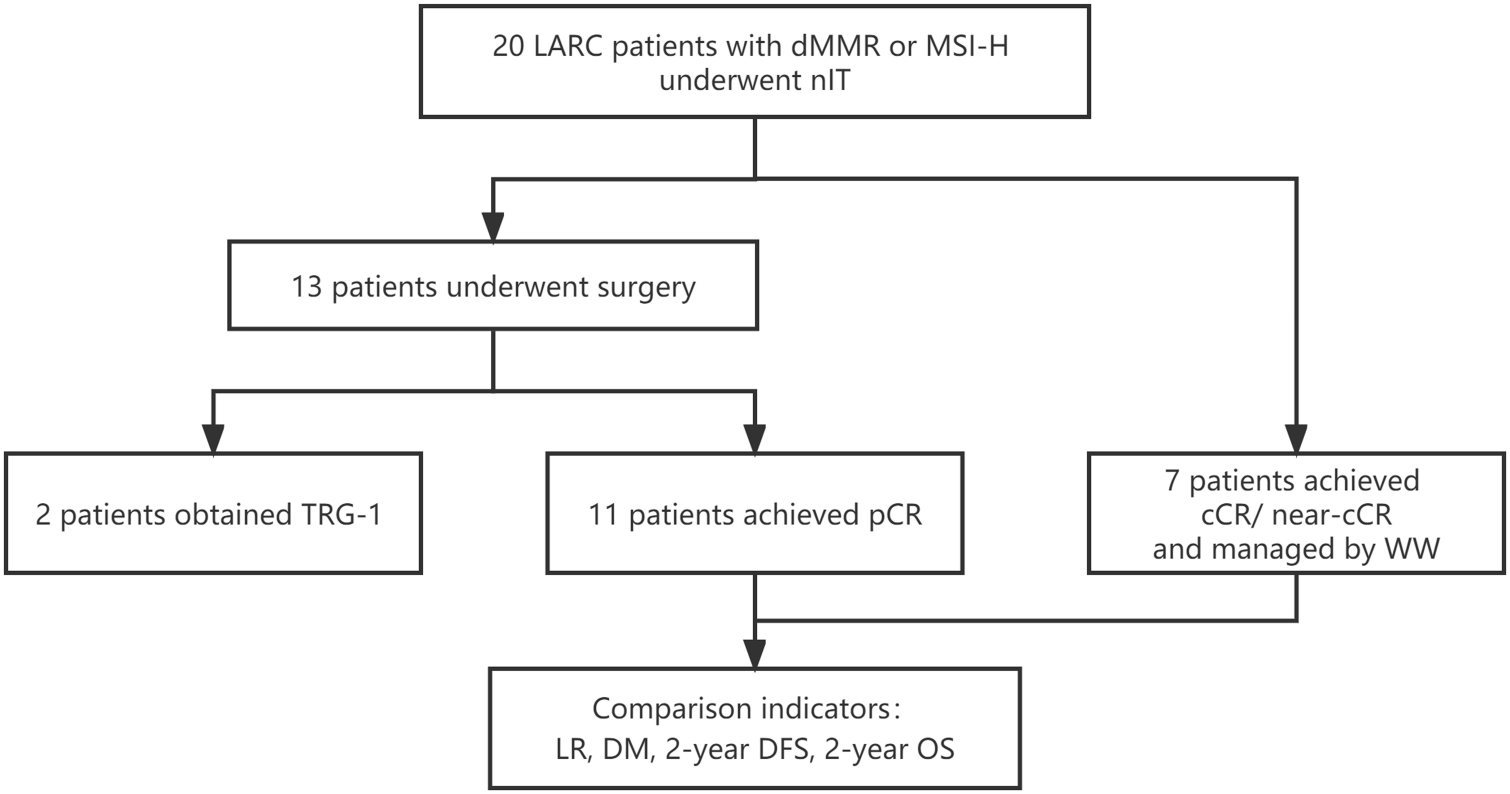
Study profile of nIT in LARC patients with dMMR/MSI-H. cCR, clinical complete response; DFS, disease-free survival; DM, distant metastasis; dMMR, mismatch repair-deficient; LARC, locally advanced rectal cancer; LR, local recurrence; MSI-H, microsatellite instability-high; nIT, neoadjuvant immunotherapy; near-cCR, near clinical complete response; OS, overall survival; pCR, pathological complete response; TRG, tumor regression grade; WW, watch-and-wait.

We identified 20 LARC patients with dMMR/MSI-H who received nIT in one of the four participating institutions from January 2019 to May 2020 (**Figure 1**). Three patients who achieved cCR and 4 patients who achieved near-cCR were in the WW group and the other 13 patients received TME. Among the 13 patients who underwent TME surgery, 11 patients achieved pCR and were in the pCR group, the other 2 patients had TRG-1 and were excluded from the comparative analysis of LR, DM, 2-year DFS, and 2-year OS.

We compared the baseline demographic and clinicopathological characteristics of all 20 patients, the 11 patients in the pCR group, and the 7 patients in the WW group (**Table 1**). We also recorded the individual details of each patient in the TME group (**Table 2**) and the WW group (**Table 3**). Patients in the pCR and WW groups were similar in terms of age, gender, ECOG performance status, personal or family history of cancer, distance of tumor from the anal verge before nIT, cT stage, cN stage, cTNM stage, maximum diameter of the primary tumor on MRI, serum CEA level before nIT, anal complex invasion, mesorectal fascia invasion (MRF+), extramural vascular invasion (EMVI+), and degree of tissue differentiation (all *P* > 0.05). The median age of all 20 patients was 55 years old (range: 23–74), and the median distance of the tumor from the anal verge before nIT was 6.5 cm. Patients in the WW group were slightly older (55 *vs.* 44 years, *P* = 0.347) and had tumors that were located closer to the anal verge (5 *vs.* 7 cm, *P* = 0.052), although these differences were not significant.

**Table 1:** Baseline demographic and clinical characteristics of patients with dMMR/MSI-H LARC.

Characteristics	Total (n=20)	pCR group (n=11)	WW group (n=7)	P-Value
Age, years				0.347
Median (range)	55 (23–74)	44(23-69)	55(43-62)	
Sex				0.141
Female	7/20(35.0%)	2/11(18.2%)	4/7(57.1%)	
Male	13/20(65.0%)	9/11(81.8%)	3/7(42.9%)	
ECOG performance status				0.627
0	11/20(55.0%)	3/11(27.3%)	3/7(42.9%)	
1	9/20(45.0%)	8/11(72.7%)	4/7(57.1%)	
Personal or family history of cancer				
Personal history of gastrointestinal Cancer	2/20(10.0%)	0	2/7(28.6%)	0.263
Personal history of extra-intestinal cancer	2/20(10.0%)	1/11(9.1%)	1/7(14.3%)	
Family history of gastrointestinal Cancer	3/20(15.0%)	2/11(18.2%)	1/7(14.3%)	0.232
Family history of extra-intestinal cancer	3/20(15.0%)	0	2/7(28.6%)	
Distance from the anal verge before nIT (cm)				
Median (range)	6.5(1-15)	7(3-15)	5(1-8)	0.052
0-5 (including 5)	8/20(40.0%)	3/11(27.3%)	4/7(57.1%)	0.304
5-10 (including 10)	9/20(45.0%)	5/11(45.5%)	3/7(42.9%)	
10-15 (including 15)	3/20(15.0%)	3/11(27.3%)	0	
cT stage				0.627
T3	7/20(35.0%)	3/11(27.3%)	3/7(42.9%)	
T4	13/20(65.0%)	8/11(72.7%)	4/7(57.1%)	
cN stage				0.205
N0	6/20(30.0%)	2/11(18.2%)	2/7(28.6%)	
N1	4/20(20.0%)	1/11(9.1%)	3/7(42.9%)	
N2	10/20(50.0%)	8/11(72.7%)	2/7(28.6%)	
cTNM stage				>0.999
II	6/20(30.0%)	2/11(18.2%)	2/7(28.6%)	
III	14/20(70.0%)	9/11(81.8%)	5/7(71.4%)	
Anal sphincter complex				0.528
Involved	4/20(20.0%)	1/11(9.1%)	2/7(28.6%)	
Uninvolved	16/20(80.0%)	10/11(90.9%)	5/7(71.4%)	
MRF				0.627
Positive	8/20(40.0%)	3/11(27.3%)	3/7(42.9%)	
Negative	12/20(60.0%)	8/11(72.7%)	4/7(57.1%)	
EMVI				0.596
Positive	6/20(30.0%)	4/11(36.4%)	1/7(14.3%)	
Negative	14/20(70.0%)	7/11(63.6%)	6/7(85.7%)	
LLNM				0.316
Yes	8/20(40.0%)	5/11(45.5%)	1/7(14.3%)	
No	12/20(60.0%)	6/11(54.5%)	6/7(85.7%)	
Maximum diameter of primary tumor on MRI before nIT (cm)				
Median (range)	5.5(2.5-15)	6.2(2.6-15)	4.5(2.5-6.9)	0.060
Serum CEA level before nIT (ug/L)				0.449
Median (range)	4.50 (1.07-272.10)	5.05 (2.42-272.10)	3.05 (1.07-48.56)	
Histological appearance				0.593
Well differentiated	7/20(35.0%)	5/11(45.5%)	2/7(28.6%)	
Moderately differentiated	5/20(25.0%)	2/11(18.2%)	3/7(42.9%)	
Poorly differentiated	8/20(40.0%)	4/11(36.4%)	2/7(28.6%)	
Loss of expression of MMR proteins				0.819
MLH1 only	2/20(10.0%)	1/11(9.1%)	1/7(14.3%)	
MSH2 only	3/20(15.0%)	2/11(18.2%)	1/7(14.3%)	
MSH6 only	0	0	0	
PMS2 only	1/20(5.0%)	0	1/7(14.3%)	
MLH1 and PMS2	4/20(20.0%)	2/11(18.2%)	0	
MSH2 and MSH6	3/20(15.0%)	2/11(18.2%)	1/7(14.3%)	
MSH2, MSH6 and PMS2	1/20(5.0%)	0	1/7(14.3%)	
Not tested	6/20(30.0%)	4/11(36.4%)	2/7(28.6%)	
MSI status				
MSI-H	10/20(50.0%)	6/11(54.5%)	4/7(57.1%)	
Not tested	10/20(50.0%)	5/11(45.5%)	3/7(42.9%)	
LS				0.596
Yes	2/20(10.0%)	1/11(9.1%)	1/7(14.3%)	
Suspected	3/20(15.0%)	1/11(9.1%)	2/7(28.6%)	
Unknown	15/20(75.0%)	9/11(81.8%)	4/7(57.1%)	
Types of PD-1 inhibitors				0.566
Tislelizumab	8/20(40.0%)	6/11(54.5%)	2/7(28.6%)	
Sintilimab	9/20(45.0%)	3/11(27.3%)	4/7(57.1%)	
Pembrolizumab	3/20(15.0%)	2/11(18.2%)	1/7(14.3%)	
Course of nIT				
Median (range)	6(4-10)	6(6-10)	8(6-10)	0.408
Efficacy evaluation after nIT				
cCR	3/20(15.0%)	0	3/7(42.9%)	
near-cCR	8/20(40.0%)	4/11(36.4%)	4/7(57.1%)	
PR	9/20(45.0%)	7/11(63.6%)	0	
Percentage of primary tumor regression after nIT (%)				0.028
Median (range)	77.53 (40-100)	69.09 (40-100)	88.89 (77.27-100)	
Adjuvant immunotherapy (aIT)				
Yes	9/20(45.0%)	7/11(63.6%)	/	
No	4/20(20.0%)	4/11(36.4%)	/	
Course of immunotherapy (nIT+aIT)				0.527
Median (range)	8(6-12)	8(6-12)	8(6-10)	

aIT, adjuvant immunotherapy; cTNM: clinical tumor node metastasis; cCR, clinical complete response; CEA, carcinoembryonic antigen; CR, complete response; CRC, colorectal cancer; dMMR, mismatch repair-deficient; ECOG, eastern cooperative oncology group; EMVI, extramural vascular invasion; LARC, locally advanced rectal cancer; LLNM, lateral lymph node metastasis; MMR, mismatch repair; MRF, mesorectal fascia; MRI, magnetic resonance imaging; MSI-H, microsatellite instability-high; near-cCR, near clinical complete response; nIT, neoadjuvant immunotherapy; pCR, pathological complete response; PR, partial response; RECIST v1.1, response evaluation criteria in solid tumors version 1.1; WW, watch-and-wait.

**Table 2:** Details of dMMR/MSI-H LARC patients treated with nIT and surgery.

Patient No. (Sex, Age, years)	cTNM stage,	MRF, EMVI	Distance from the anal verge (cm)	Personal or family history of cancer	LS	Loss of MMR protein expressions	MS	Maximum diameter of primary tumor on MRI pre- and post- nIT (cm)	Regimen of nIT	Courses of nIT	Serum CEA level pre- and post- nIT (ug/L)	Response on DRE	Response on endoscopic, and biopsy	Response on MRI, and TRUS	Response evaluation based on the RECIST v1.1	Surgical approach	ypTNM stage, TRG	Courses of aIT
1 (M, 23y)	cT4aN2M0	Positive, Positive	5	Yes	Yes	MSH2, MSH6	MSI-H	11.0, 3.4	Tislelizumab 200mg, q3w	10	8.82, 2.88	Scar	CR, Negative for tumor cell	PR, PR	PR	LAR with a temporary ileostomy	ypT0N0M0, 0	0
2 (M, 55y)	cT4aN2M0	Negative, Positive	2	No	Unknown	MLH1	MSI-H	7.2, 2.7	Tislelizumab 200mg, q3w	6	2.68, 3.43	Palpable tumor	PR, Not tested	PR, Not tested	PR	APR	ypT0N0M0, 0	0
3 (F, 37y)	cT4bN2M0	Negative, Negative	7	Yes	Suspected	Not tested	MSI-H	15.0, 2.0	Sintilimab 200mg, q3w	10	272.1, 2.88	Palpable tumor	PR, Negative for tumor cell	PR, PR	PR	AR	ypT0N0M0, 0	2
4 (M, 40y)	cT4bN2M0	Negative, Negative	14	No	Unknown	Not tested	MSI-H	6.2, 0	Sintilimab 200mg, q3w	10	6.08, 3.29	Scar	CR, Negative for tumor cell	CR, Not tested	cCR	AR	ypT0N0M0, 0	0
5 (M, 62y)	cT3N1M0	Negative, Negative	5	No	Unknown	Not tested	MSI-H	7.6, 0.9	Tislelizumab 200mg, q3w	8	5.05, 1.89	Scar	CR, Negative for tumor cell	near-CR, near-CR	near-cCR	LAR with a temporary ileostomy	ypT0N0M0, 0	2
6 (M, 35y)	cT4bN2M0	Negative, Negative	15	No	Unknown	MLH1, PMS2	Not tested	13.6, 1.1	Pembrolizumab 200mg, q3w	6	3.72, 2.17	Smooth mucosa	CR, Negative for tumor cell	near-cCR, Not tested	near-cCR	AR	ypT0N0M0, 0	2
7 (F, 44y)	cT4aN2M0	Positive, Negative	10	No	Unknown	MLH1, PMS2	Not tested	6.1, 2.3	Sintilimab 200mg, q3w	6	3.16, 0.91	Scar	CR, Negative for tumor cell	near-cCR, near-cCR	near-cCR	AR	ypT0N0M0, 0	2
8 (M, 67y)	cT4bN2M0	Negative, Negative	12	No	Unknown	MSH2, MSH6	Not tested	7.5, 3.7	Pembrolizumab 200mg, q3w	6	39.0, 1.76	Palpable tumor	PR, Not tested	PR, Not tested	PR	AR	ypT0N0M0, 0	0
9 (M, 69y)	cT4bN0M0	Negative, Positive	5	No	Unknown	Not tested	MSI-H	5.0, 1.3	Tislelizumab 200mg, q3w	6	28.02, 3.12	Palpable tumor	PR, Negative for tumor cell	PR, PR	PR	LAR with a temporary ileostomy	ypT0N0M0, 0	2
10 (M, 40y)	cT3N0M0	Negative, Negative	3	No	Unknown	MSH2	Not tested	3.0, 1.8	Tislelizumab 200mg, q3w	6	4.99, 4.6	Palpable tumor	PR, Negative for tumor cell	PR, PR	PR	APR	ypT0N0M0, 0	2
11 (M, 59y)	cT3N2M0	Positive, Positive	7	Yes	Unknown	MSH2	Not tested	5.7, 1.9	Tislelizumab 200mg, q3w	6	2.42, 0.71	Palpable tumor	PR, Negative for tumor cell	PR, PR	PR	AR	ypT0N0M0, 0	2
① (M, 74y)	cT3N0M0	Positive, Positive	8	No	Unknown	MLH1, PMS2	Not tested	5.5, 2.8	Sintilimab 200mg, q3w	4	2.23, 1.57	Palpable tumor	PR, Negative for tumor cell	PR, PR	PR	AR	ypT1N0M0, 1	4
② (F, 57y)	cT4bN0M0	Negative, Positive	10	Yes	Unknown	MLH1, PMS2	Not tested	9.2, 3.6	Sintilimab 200mg, q3w	6	4.00, 3.47	Palpable tumor	PR, Adenocarcinoma	PR, PR	PR	AR	ypT1N0M0, 1	2

Patient No. 1-11, the patients in the pCR group; ①-②, the patients who did not achieved pCR.

aIT, adjuvant immunotherapy; AR, anterior resection; APR, abdominoperineal resection; cCR, clinical complete response; CEA, carcinoembryonic antigen; CR, complete response; dMMR, mismatch repair-deficient; DRE, digital rectal examination; EMVI, extramural vascular invasion; LAR, low anterior resection; LARC, locally advanced rectal cancer; LS, lynch syndrome; MMR, mismatch repair; MRF, mesorectal fascia; MRI, magnetic resonance imaging; MS, Microsatellite status; MSI-H, microsatellite instability-high; nIT, neoadjuvant immunotherapy; PR, partial response; RECIST v1.1, response evaluation criteria in solid tumors version 1.1; TNM, tumor Node Metastasis; TRG, tumor regression grade; TRUS, transrectal ultrasound; ypTNM stage, pathological tumor node metastasis staging after neoadjuvant therapy.

**Table 3:** Details of dMMR/MSI-H LARC patients treated with nIT and WW strategy.

Patient No. (Sex, Age, years)	cTNM stage	MRF, EMVI,	Distance from the anal verge (cm)	Personal or family history of cancer	LS	Loss of MMR protein expression	MS	Maximum diameter of primary tumor on MRI pre- and post- nIT(cm)	Regimen of nIT	Courses of nIT before taking WW	Serum CEA level pre- and post- nIT (ug/L)	Response on DRE	Response on endoscopic, and biopsy	Response on rectal MRI, and TRUS	Response evaluated based on the RECIST v1.1
I (M, 58y)	cT4bN0M0	Negative, Negative	8	No	Unknown	MSH2, MSH6, PMS2	Not tested	8.1, 0	Sintilimab 200mg, q3w	10	8.8, 1.28	Scar	CR, Negative for tumor cell	CR, CR	cCR
II (M, 62y)	cT3N1M0	Positive, Negative	2	No	Unknown	Not tested	MSI-H	4.3, 0	Sintilimab 200mg, q3w	8	1.37, 0.86	Scar	CR, Negative for tumor cell	CR, CR	cCR
III (F, 50y)	cT4aN1M0	Negative, Negative	8	Yes	Yes	Not tested	MSI-H	2.7, 0	Tislelizumab 200mg, q3w	8	1.07, 1.03	Smooth mucosa	CR, Negative for tumor cell	CR, CR	cCR
IV (F, 61y)	cT3N1M0	Negative, Negative	1	No	Unknown	PMS2	Not tested	2.2, 0.5	Sintilimab 200mg, q3w	8	3.05, 1.94	Scar	CR, Negative for tumor cell	near-CR, CR	near-cCR
V (F, 55y)	cT3N0M0	Negative, Negative	6	Yes	Suspected	MSH2	MSI-H	6.1, 0.7	Pembrolizumab 200mg, q3w	6	1.47, 0.98	Scar	CR, Negative for tumor cell	near-CR, near-CR	near-cCR
VI (M, 43y)	cT4bN2M0	Positive, Positive	2	Yes	Suspected	MSH2, MSH6	MSI-H	9.4, 0.9	Tislelizumab 200mg, q3w	6	48.56, 0.74	Smooth mucosa	near-CR, Negative for tumor cell	near-CR, near-CR	near-cCR
VII (F, 50y)	cT4bN2M0	Positive, Negative	5	No	Unknown	MLH1	Not tested	4.5, 1.0	Sintilimab 200mg, q3w	10	7.00, 2.58	Scar	CR, Negative for tumor cell	near-CR, near-CR	near-cCR

cCR, clinical complete response; cTNM, clinical tumor node metastasis; CEA, carcinoembryonic antigen; CR, complete response; dMMR, mismatch repair-deficient; DRE, digital rectal examination; EMVI, extramural vascular invasion; LARC, locally advanced rectal cancer; LS, lynch syndrome; MMR, mismatch repair; MRF, mesorectal fascia; MRI, magnetic resonance imaging; MS, Microsatellite status; MSI-H, microsatellite instability-high; nIT, neoadjuvant immunotherapy; PR, partial response; RECIST v1.1, response evaluation criteria in solid tumors version 1.1; TRG, tumor regression grade; TRUS, transrectal ultrasound.

Among all 20 patients, 7 had a family or personal history of malignancy. In the latter group, the 3 patients with a personal and family history of cancer and the 2 with only a family history of gastrointestinal malignancy were suspected to have Lynch syndrome, but only 2 of them received tests for the relevant germline genes and had confirmed Lynch syndrome: patient III in the WW group (**Table 3**) and patient 1 in the pCR group (**Table 3**). Patient III in the WW group received surgery and chemotherapy for jejunal well-differentiated adenocarcinoma at the age of 30s and ovarian clear cell carcinoma at the age of 40s, and the mother of this patient died from ovarian cancer; genetic testing results indicated this patient had an exon 1 germline mutation in the *MLH1* gene. Patient 1 in the pCR group had a grandmother who died from gastric cancer, and the father of this patient had rectal cancer at the time of this study; genetic testing identified a germline mutation in exon 7 of *MSH2* gene.

Analysis of microsatellite status indicated 1 patient had MSI-H based on PCR, and 5 patients had MSI-H based on NGS. IHC results on pre-treatment tumor specimens in 10 patients confirmed dMMR status, and the remaining 4 patients had both dMMR (by IHC) and MSI-H (by NGS or PCR). Among the 14 patients identified as dMMR, one had losses of MSH2, MSH6, and PMS2; 4 had losses of MLH1 and PMS2; 3 had losses of MSH2 and MSH6; 2 had a loss of MLH1; 1 had a loss of PMS2; and 3 had a loss of MSH2. The pCR and WW groups had no statistically significant difference in MMR protein deletions (*P* = 0.819).

### Efficacy of nIT with PD-1 inhibitors and adjuvant therapy

**Figure 2:**
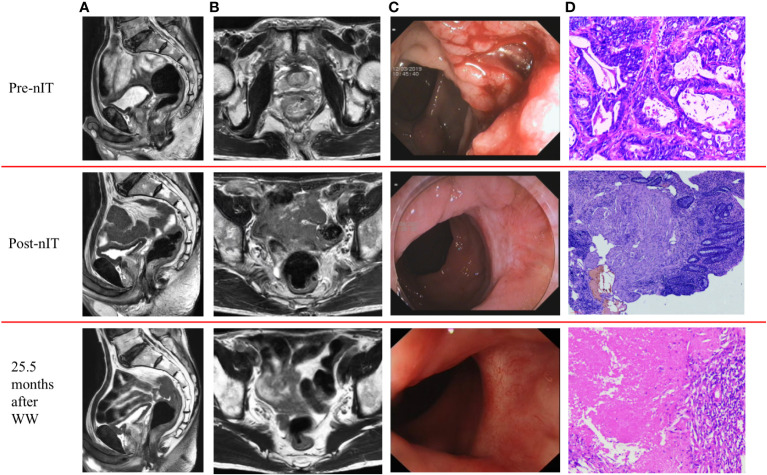
Representative radiologic, colonoscopic and pathological response to nIT in one patient with cCR (patient II in **Table 3**). **(A)** Sagittal plane MR views of the pelvis: pre-nIT VS post-nIT VS 25.5 months after WW; **(B)** Axial plane MR views of the pelvis: pre-nIT VS post-nIT VS 25.5 months after WW; **(C)** Colonoscopy: pre-nIT VS post-nIT VS 25.5 months after WW; **(D)** Pathology: tumor biospy of pre-nIT (HE x40) VS re-biospy of post-nIT (HE x40) VS re-biospy of 25.5 months after WW (HE x40). cCR, clinical complete response; HE, hematoxylin-eosin; MR, Magnetic resonance; nIT, neoadjuvant immunotherapy.

**Figure 3:**
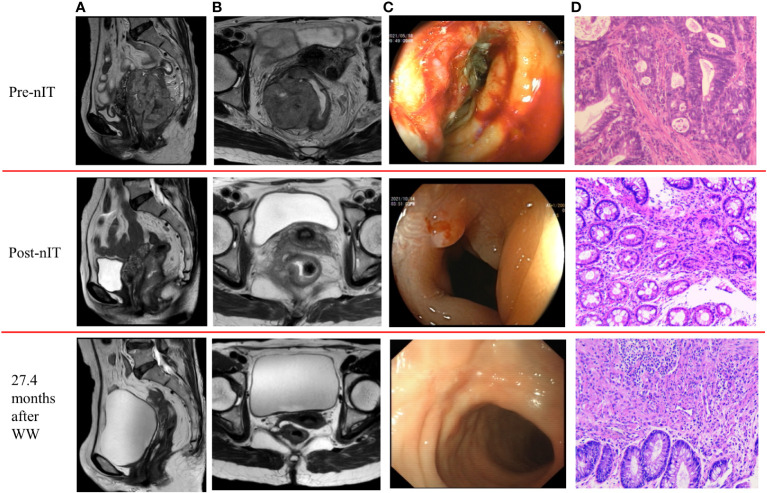
Representative radiologic, colonoscopic and pathological response to nIT in one patient with near-cCR (patient VI in **Table 3**). **(A)** Sagittal plane MR views of the pelvis: pre-nIT VS post-nIT VS 27.4 months after WW; **(B)** Axial plane MR views of the pelvis: pre-nIT VS post-nIT VS 27.4 months after WW; **(C)** Colonoscopy: pre-nIT VS post-nIT VS 27.4 months after WW; **(D)** Pathology: tumor biospy of pre-nIT (HE x40) VS re-biospy of post-nIT (HE x40) VS re-biospy of 27.4 months after WW (HE x40). HE, hematoxylin-eosin; MR, magnetic resonance; nIT, neoadjuvant immunotherapy;near-cCR, near clinical complete response.

All 20 patients received PD-1 inhibitor monotherapy as the initial treatment (8 patients with tislelizumab, 9 with sintilimab, and 3 with pembrolizumab) and there were no significant differences in the type of drug used in the WW and the pCR groups (**Table 1**). After completing a median of 6 cycles (range: 4–10) of nIT, the objective response rate (ORR) was 100% (20/20), the cCR rate was 15% (3/20), the near-cCR rate was 40.0% (8/20), and the partial response (PR) rate was 45.0% (9/20) (**Table 1**). Representative MRI, endoscopic and pathological images of patients with dMMR/MSI-H LARC who achieved cCR and near-cCR after nIT are shown in **Figures 2**, **3**.

**Figure 4:**
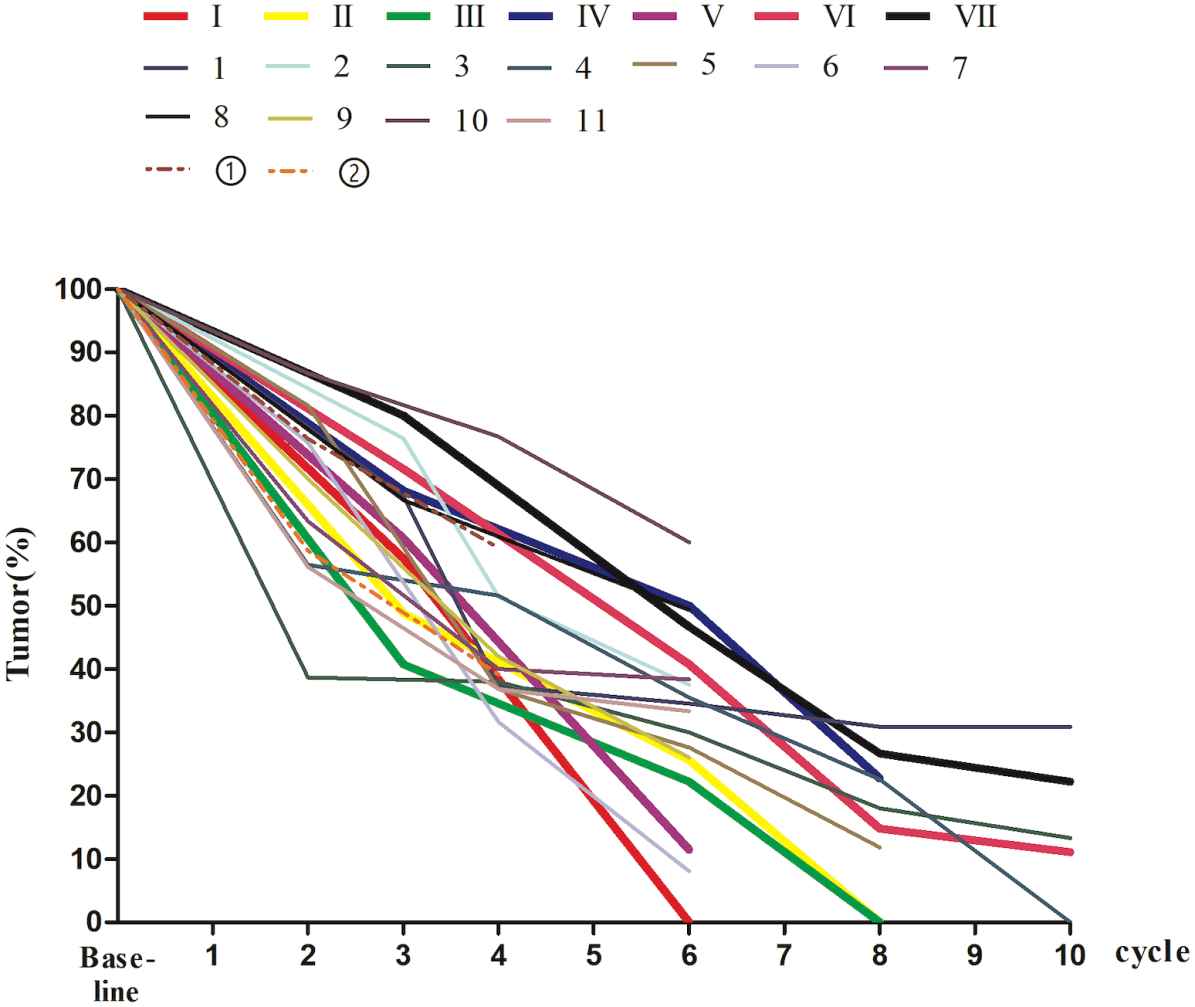
The percentage of tumor size on MRI at baseline and during nIT in 20 dMMR/MSI-H LARC patients. I-VII, the patients in the WW group; 1-11, the patients in the pCR group; ①-②, the patients who did not achieved pCR. dMMR, mismatch repair-deficient; LARC, locally advanced rectal cancer; MRI, magnetic resonance imaging; MSI-H, microsatellite instability-high; nIT, neoadjuvant immunotherapy; pCR, pathological complete response; WW, watch-and-wait.

The pCR and WW groups had no significant difference in the median number of cycles of nIT (6 *vs.* 8, *P* = 0.408), however, patients in the WW group exhibited greater radiographic regression in the primary lesion (*P* = 0.028; **Table 1** and **Figure 4**). For all 20 patients, the WW group, and the pCR group, there was a median of 3 cycles of nIT (range: 2–6) from treatment initiation to PR, corresponding to 2.25 months (range: 1.5–4.5); and there was a median of 8 cycles of nIT (range: 6–10) needed to achieve cCR or near-cCR.

**Figure 5:**
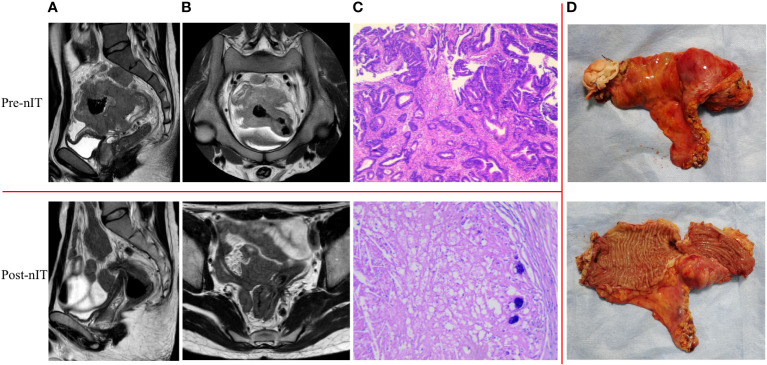
Representative radiologic, resection specimen and pathological response to nIT in one patient with pCR (patient 1 in **Table 2**). **(A)** Sagittal plane MR views of the pelvis: pre- VS post- nIT; **(B)** Axial plane MR views of the pelvis: pre- VS post- nIT; **(C)** Pathology: tumor biospy of pre-nIT (HE x40) VS postoperative specimen of post-nIT (HE x40); **(D)** Specimen: Resection specimen of post-nIT. HE, hematoxylin-eosin; MR, magnetic resonance; nIT, neoadjuvant immunotherapy; pCR, pathological complete response.

For the 13 patients who underwent TME after nIT (9 with PR, 1 with cCR, 3 with near-cCR), the ORR, pathological response rate, and MPR were all 100% (13/13), and the pCR was 84.6% (11/13). Typical images from MRI, post-nIT resection specimens, and pathologic response of patients with dMMR/MSI-H LARC who achieved a pCR are shown in **Figure 5**. The two patients who did not achieve pCR had TRG-1 and pathological stage of ypT1N0M0 (**Table 4**). Notably, 3 patients achieved a pCR after 6 to 10 cycles of nIT, despite their very large tumors (>10 cm) and very late clinical stage (cT4N2M0) (**Table 2**).

**Table 4:** Pathological outcomes of dMMR/MSI-H LARC patients treated with nIT and surgery.

Outcomes	nIT and surgery group (n=13)
ORR	13/13 (100%)
Pathological response rate	13/13 (100%)
MPR rate	13/13 (100%)
pCR rate	11/13(84.6%)
TRG	
0	11/13(84.6%)
1	2/13(15.4%)
2	0
3	0
Pathological T stage	
ypT0	11/13(84.6%)
ypT1	2/13(15.4%)
ypT2	0
ypT3	0
Pathological N stage	
ypN0	13/13 (100%)
ypN1	0
Pathological TNM stage	
ypT0N0M0	11/13(84.6%)
ypT1N0M0-I	2/13(15.4%)
ypT2N0M0-I	0
ypT3N0M0-IIA	0

dMMR, mismatch repair-deficient; LARC, locally advanced rectal cancer; MPR, major pathological response; MSI-H, microsatellite instability-high; nIT, neoadjuvant immunotherapy; ORR, objective response rate; pCR, pathological complete response; TNM, tumor Node Metastasis; TRG, tumor regression grade.

Adjuvant anti-PD-1 monotherapy was administered to 69.2% (9/13) of patients with surgery, including 7 patients with pCR and 2 with TRG-1. Most of them received 2 cycles, only 1 patient with TRG-1 who completed just 4 cycles of nIT received 4 cycles of aIT. None of the patients in the WW group continued to use PD-1 inhibitors after achieving cCR or near-cCR. There was a median of 8 cycles (6 months) of immunotherapy (nIT + aIT) in all 20 patients, in the pCR group, and in the WW group (**Table 1**).

### Treatment-related adverse events and enterostomy

We evaluated treatment-related adverse events (including those related to immunotherapy and surgery) and enterostomy (**Tables 2**, **3**). During the neoadjuvant and adjuvant phases, the incidence of irAEs was 35.0% (7/20) for all 20 patients, 45.5% (4/11) in the pCR group, and 28.6% (2/7) in the WW group (*P* = 0.627). Most of the irAEs were grade 1, but one patient developed a grade 2 hypothyroidism that was significantly relieved by low-dose thyroxine supplementation. There were no grade 3 or higher irAEs (**Table 5**).

**Table 5:** Immune-related adverse events (irAEs) of patients with dMMR/MSI-H LARC.

Adverse events	Total (n=20)			pCR group (n=11)			WW group (n=7)			
	Grade 1	Grade 2	Grade≧3	Grade 1	Grade 2	Grade≧3	Grade 1	Grade 2	Grade≧3	
irAEs										
Pneumonia	1	0	0	0	1	0	0	0	0
Hypothyroidism	0	1	0	0	0	0	0	1	0
Nausea	1	0	0	1	0	0	0	0	0
Fatigue	1	0	0	0	0	0	0	0	0
Neutropenia	1	0	0	1	0	0	0	0	0
Pruritus or rash	1	0	0	1	0	0	0	0	0
Decreased appetite	1	0	0	0	0	0	1	0	0
Bowel obstruction	0	1	0	0	1	0	0	0	0
Total	6/20 (30.0%)	2/20 (10.0%)	0	3/11 (27.3%)	2/11 (18.2%)	0	1/7 (14.3%)	1/7 (14.3%)	0	
	8/20(40.0%)			4/11(45.5%)			2/7 (28.6%)			
P-value				P=0.627					

dMMR, mismatch repair-deficient; irAEs; immune-related adverse events; LARC, locally advanced rectal cancer; MSI-H, microsatellite instability-high; pCR, pathological complete response; WW, watch-and-wait.

Among the 13 patients who received TME, the median time from the last nIT to surgery was 25 days (18–42), and none of these patients had a delay of surgery due to an irAE. All 13 of these patients achieved R0 resection: 2 (15.4%) received abdominoperineal resection (APR) and 11 (84.6%) received sphincter-saving surgery. In the latter group, 3 (23.1%) patients received low anterior resection (LAR) with a temporary ileostomy. There were srAEs in 3 (23.1%) of these patients: a grade 1 surgical incision with poor healing, a grade 1 postoperative anastomotic bleeding, and a grade 2 anastomotic leak. No patient experienced perioperative mortality or severe surgery-related morbidity requiring re-operation (**Table 6**).

**Table 6:** Surgical-related adverse events (srAEs) and enterostomy of patients with dMMR/MSI-H LARC ungerwent surgery.

Adverse events	Total (n=13)			pCR group (n=11)		
	Grade 1	Grade 2	Grade≧3	Grade 1	Grade 2	Grade≧3
srAEs						
Incision infection	1	0	0	1	0	0
Postoperative bleeding	1	0	0	0	0	0
Anastomotic leakage	0	1	0	0	1	0
Total	2/13 (15.4%)	1/13 (7.7%)	0	1/11 (9.1%)	1/11 (9.1%)	0
	3/13 (23.1%)			2/11 (18.2%)		
Enterostomy						
Temporary ileostomy	3/13 (23.1%)			3/11 (27.3%)		
Permanent colostomy	2/13 (15.4%)			2/11 (18.2%)		

dMMR, mismatch repair-deficient; LARC, locally advanced rectal cancer; MSI-H, microsatellite instability-high; pCR, pathological complete response; srAEs, surgical-related adverse events.

### Recurrence and survival outcomes

**Figure 6:**
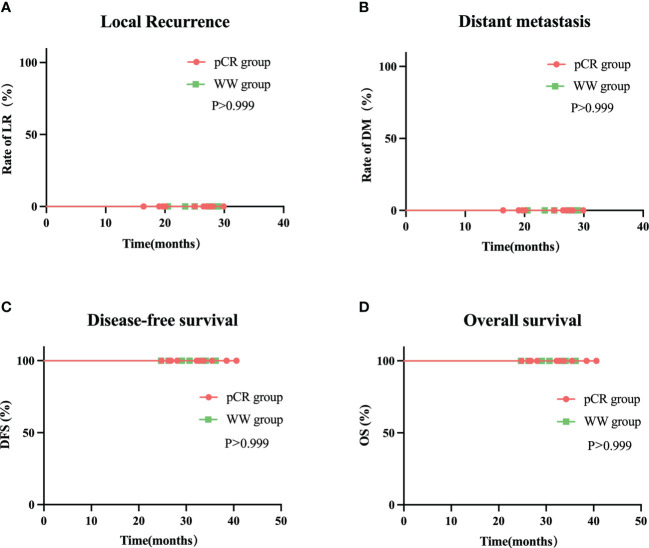
Rate of local recurrence **(A)** distant metastasis **(B)** disease-free survival **(C)** and overall survival **(D)** in WW group and pCR group during follow-up.

The median follow-up time was 24.35 months (range: 16.4–29.9) in all 20 patients, 24.5 months (range: 16.4–29.9) in the pCR group, and 25.0 months (range: 20.5–29.0) in the WW group (P = 0.633; **Table 7**). None of the 20 patients (including the 2 with TRG-1) experienced LR, DM, or death as of the last follow-up date (June 15, 2022). Thus, the pCR group and WW group had 2-year DFS rates and 2-year OS rates of 100%, and LR rates and DM rates of 0% (**Table 7** and **Figure 6**). Remarkably, 4 patients who adopted a WW strategy after achieving near-cCR and did not continue anti-PD-1 therapy did not experience LR or DM, even though some of them had high-risk factors at baseline, such as EMVI+, MRF+, and T4 stage (**Table 3**).

**Table 7:** Recurrence and survival in dMMR/MSI-H LARC patients with nIT.

Recurrence or survival	Total (n=20)	pCR group (n=11)	WW group (n=7)	P-Value
LR	0	0	0	>0.999
DM	0	0	0	>0.999
2-year DFS	20/20(100%)	11/11(100%)	7/7(100%)	>0.999
2-year OS	20/20(100%)	11/11(100%)	7/7(100%)	>0.999
Median Follow-up (months)	24.35 (16.4-29.9)	24.50 (16.4-29.9)	25.00 (20.5-29.0)	0.633

DFS, disease-free survival; DM, distant metastasis; dMMR, mismatch repair-deficient; LARC, locally advanced rectal cancer; LR, local recurrence; MSI-H, microsatellite instability-high; nIT, neoadjuvant immunotherapy; OS, overall survival; pCR, pathological complete response; WW, watch-and-wait.

## Discussion

Our real-world study examined a multicenter cohort of LARC patients with dMMR/MSI-H who were treated with neoadjuvant PD-1 inhibitor monotherapy. During the 2-year follow-up period, 100% (7/7) of patients who were managed with a WW strategy after achieving a cCR or near-cCR had a comparable oncological safety profile as those who accepted TME surgery. More importantly, patients in the WW group did not experience a reduced quality of life associated with surgical complications, enterostomies, or deterioration of the bowel, urinary system, or sexual function. Moreover, in the WW group, the 4 patients with near-cCR achieved the same rate of organ sparing and oncological safety as the other 3 patients with cCR (**Table 3**), similar to the results of the OPERA study (11). Thus, for LARC patients with dMMR/MSI-H who achieve cCR or near-cCR after nIT, the WW strategy is a safe and beneficial option.

Previous studies indicated that the 2-year LR rate of LARC patients who achieved cCR and adopted a WW strategy after nCRT was 19 to 25% (9, 34). In our study, a WW approach, even for patients with near-cCR or high-risk factors for LR and DM (EMVI+, MRF+, or T4 stage), led to a 2-year LR rate and DM rate of 0%. This remarkable efficacy may be because the nIT can convert the high level of tumor antigens produced by the primary tumor with dMMR/MSI-H into “autologous vaccines”, which activate and recruit more tumor-specific T cells and promote the formation of long-term immune memory, rather than simply killing tumor cells. This kind of systemic and persistent immunity against tumors enables greater clearance of micrometastases that cannot be eliminated by surgery or radiotherapy and reduces the rate of LR and DM (35–37).

The WW strategy described herein is indeed promising, but accurate assessment of cCR and near-cCR remains a difficult problem. Due to the lack of more sensitive assessment methods, we used the same diagnostic criteria for cCR and near-cCR as used in patients after traditional nCRT. However, the mechanism of action and response of immunotherapy are markedly different from those of conventional therapy with cytotoxic drugs and radiation (38). These could lead to inconsistent clinical and pathological evaluations, as indicated by an underestimated efficacy of immunotherapy based on imaging. In this study, although the imaging results demonstrated that the 3 patients with near-cCR and the 7 patients with PR in the pCR group still had residual mass, the pathology results revealed that none of their surgical resection specimens had residual tumor cells, but instead consisted of massive inflammatory cells or mucus lakes (**Table 2**). The recent PICC study found that 28 of 35 patients who were evaluated as PR preoperatively had pCR based on postoperative pathology (23). This inconsistency also occurred in studies of nIT for other solid malignancies (39, 40). A possible explanation is that radiology cannot easily distinguish masses consisting of inflammatory cells, necrotic tissue, and/or fibrous tissues from masses consisting of tumor cells. This issue appears to challenge the routine use of established morphological-based response evaluation criteria.

Fortunately, tests other than conventional imaging examinations have gradually been used for efficacy evaluation in patients with malignancy. For example, ^18^F-fluorodeoxyglucose-positron emission computed tomography (^18^F-FDG-PET/CT), a valuable tool that combines anatomic morphologic imaging with functional metabolic imaging, can help to distinguish malignant and non-malignant masses. Goldfarb et al. first proposed the immune PET response criteria in solid tumors (iPERCIST) in 2019. They reported that this criterion could compensate for about 39% of the response underestimated by the RECIST 1.1 criteria in non-small cell lung cancer patients who received ICIs (41). In the 2022 study of Cercek et al., 12 rectal cancer patients with dMMR/MSI-H who were evaluated as cCR by traditional anatomical imaging combined with ^18^F-FDG-PET/CT after 6 months of neoadjuvant dostarlimab treatment and adopted a WW strategy did not develop recurrence during the 6 to 25-month follow-up period (21). In addition, other studies found that changes in the level of circulating tumor DNA (ctDNA) could be used to predict response to immunotherapy (42, 43). Bratman et al. reported a 100% OS rate of patients with solid tumors who had ctDNA elimination after pembrolizumab treatment during a median follow-up time of 25.4 months (43). Hence, a regimen that combines radiological, metabolic, and hematological parameters might improve the accuracy of the efficacy evaluations of immunotherapy, and this could facilitate early identification of patients who would benefit most from a WW strategy and prevent over-treatment.

Furthermore, an underestimation of the efficacy of immunotherapy from imaging suggests that rectal cancer patients with dMMR/MSI-H who were treated with ICIs may have achieved pCR before being evaluated as near-cCR, cCR, or even PR. Another consideration is that patients who receive immunotherapy have unique remission patterns, such as delayed response, pseudo-progression (38), and long-lasting efficacy. We therefore suggest that LARC patients who are considering a WW strategy after nIT — even if imaging does not yet indicate cCR — should be offered the option of more time for observations or more cycles of ICIs followed by re-evaluation, rather than early surgical resection. Transanal local tumor resection, another organ-sparing option, may be preferred to proctectomy for rectal cancer patients who have a persistent clinical stage of ycT1N0M0 after nIT.

In terms of optimal efficacy, our results demonstrated a 90% CR rate (55% pCR, 15% cCR, and 20% near-cCR) was achieved after 6 cycles (range 4–10) of a neoadjuvant PD-1 inhibitor. This is better than reported in the NICHE study (17), the NICHE-2 study (18), and the PICC study (23), but worse than in the study of Cercek et al. (21). We believe that these differences in efficacy can be partly explained by differences in the dose and duration of the ICIs. In particular, the NICHE study and NICHE-2 study reported pCR rates of dMMR/MSI-H colon cancer patients were 69% and 67% after one dose of ipilimumab (1 mg/kg on day 1) and two doses of nivolumab (3 mg/kg on days 1 and 15). The PICC study reported the pCR rate in LARC patients with dMMR/MSI-H after 6 cycles of toripalimab (3 mg/kg) was 88% with celecoxib and 65% without celecoxib. However, the Cercek et al. study of LARC patients with dMMR/MSI-H reported the cCR rate was 100% after neoadjuvant treatment with 9 cycles of dostarlimab (500 mg every 3 weeks for 6 months).

Another consideration is that these previous studies examined tumors at different sites. The Cercek et al. (21). study and our study examined LARC patients and achieved CR rates of 90% or more; this is higher than in the NICHE study (17) and the NICHE-2 study (18), which examined patients with colon cancer. The PICC study reported a slightly higher pCR rate in rectal cancer patients (83.33%) than in colon cancer patients (78.57%) (23), and Liu et al. reported similar results (pCR rate, rectal cancer: 100%, colon cancer: 50%) (44). These differences in efficacy suggest that rectal cancer patients with dMMR/MSI-H appear to benefit more from nIT than colon cancer patients with dMMR/MSI-H. There is also evidence of differences in the distribution of gut microbiota in different parts of the colorectum. In particular, rectal cancer patients have significantly more diverse gut microbiota than colon cancer patients (45, 46). Furthermore, several gut microbes can affect the efficacy of ICIs (39, 47), such as *Fusobacterium nucleatum*, which induces various immune responses in CRCs with distinct microsatellite states, and can enhance the efficacy of ICIs (48, 49).

However, LARC patients with dMMR/MSI-H achieved a 75% CR rate (lower than our study) after a median of 8 cycles of nIT with sintilimab (higher than our study), and a case of primary resistance to ICIs was reported in a recent study (50). It indicates that factors other than those mentioned above influence patient efficacy, among which tumor mutation burden (TMB), gene status and immune microenvironment are several research hotspots. For instance, dMMR/MSI-H CRC patients with low TMB, immunosuppressive tumor microenvironment, or mutation in the PTEN gene or PIK3CA gene are resistant to ICIs (51, 52), while pMMR/MSS CRC patients with a mutation in POLE or POLD1 gene are sensitive to ICIs (53). Therefore, despite nIT being highly effective for CRC patients with dMMR/MSI-H, the inclusion of the analysis of the molecular profile as well as the immune contexture remains imperative.

It is currently uncertain whether continuous ICI treatment is required for CRC patients who achieve a cCR or pCR after nIT. In the NICHE study (17) and the NICHE-2 study (18), aIT was not offered to 69% (22/32) and 67% (72/107) of dMMR colon cancer patients who achieved pCR. The 12 LARC patients with dMMR/MSI-H in the study of Cercek et al. (21). who achieved a cCR also did not continue ICI treatment after adopting a WW strategy. Notably, patients in all three previous studies who attained pCR or cCR did not develop LR or DM during the follow-up period (median: 25 months for NICHE, 13 months for NICHE-2, and 12 months for Cercek et al.). However, the PICC study recommended adjuvant use of toripalimab with or without celecoxib for all patients, regardless of whether they attained pCR, until completion of 6 months of perioperative anti-PD-1 therapy (23). In our study, the median time from treatment initiation to CR was 5.25 months (range 4.5–7.5), the median time of the entire perioperative period was 6 months (range 4.5–9.0), and we observed no recurrence or metastasis during the 2-year follow-up. The value of aIT is currently unknown, indicating the need for large prospective studies.

Several studies reported that ctDNA is associated with an increased risk of recurrence (54, 55). This ctDNA reflects minimal residual disease (MRD), which is responsible for the most postoperative recurrences (56). Henriksen et al. studied patients with stage III colon cancer and compared ctDNA-negative and ctDNA-positive patients after surgery and adjuvant chemotherapy. They found a 7-fold increased risk of recurrence for patients who were ctDNA-positive after surgery and a 50-fold increased risk of recurrence for patients who were ctDNA-positive after adjuvant therapy, while patients with continuous negative ctDNA during and after adjuvant therapy had no recurrence (57). Therefore, a ctDNA-based MRD assay may help to identify patients with high risk of relapse, and provide more personalized suggestions for adjuvant treatment and surveillance (54, 57). Three ongoing prospective clinical studies (NCT04761783; NCT05198154; NCT04636047) are examining the use of ctDNA-based MRD assays for guiding immunotherapy.

Finally, nIT for LARC is still in its infancy, and there is no unified standard regarding the therapeutic dose, course, efficacy evaluation criteria, or the need for aIT. Although outcomes appear promising, this study was limited by its retrospective design, small sample size, and short follow-up time. Firstly, a WW strategy after obtaining cCR or near-cCR following nIT is not currently a standard therapy, and the decision to circumvent proctectomy was mostly driven by an individual patient’s strong desire for preservation of organ function and avoidance of enterostomy. This is why there were only 7 cases in our WW group. Secondly, the median follow-up time of our study was only 24.35 months. However, this was the longest follow-up time of any study that examined the WW strategy after nIT. In addition, due to economic and other reasons, only two patients in our study had a detection of germline genes and confirmed a diagnosis of Lynch syndrome. Given the familial heritability of Lynch syndrome and the potential for multiple primary malignancies, we will continue to encourage subjects who are young or suspected of Lynch syndrome to perform germline genetic testing for better long-term management and follow-up.

## Conclusions

In conclusion, our study verified the feasibility and safety of a WW strategy for LARC patients with dMMR/MSI-H who achieved cCR or near-cCR after nIT. Our results suggest that a WW strategy for these patients could help to preserve sphincter function and improve long-term survival. Longer follow-up studies and prospective trials are needed to evaluate this promising treatment option.

## Data availability statement

All datasets supporting the conclusions of this article are included within the article and its **Supplementary files**.The datasets used during the current study are available from the corresponding author on reasonable request.

## Ethics statement

This retrospective study was conducted according to the Declaration of Helsinki, and approved to waive informed patient consent by the ethics review boards of the Yunnan Cancer Hospital (Third Affiliated Hospital of Kunming Medical University) (approval number KYLX2022053) because it was an observational and non-interventional study.

## Author contributions

Drafting the work and/or revising it critically: RY, TW and JY. Final approval of the version to be published: PD, XZ, YL, and RY. All authors contributed to the article and approved the submitted version.
