# Supplementary material for: Locally advanced rectal cancer with dMMR/MSI-H may be excused from surgery after neoadjuvant anti-PD-1 monotherapy: a multiple-center, cohort study
Source: Front Immunol. 2023 Jun 27;14:1182299. doi: 10.3389/fimmu.2023.1182299 (PMC10333582; doi:10.3389/fimmu.2023.1182299)
Supplement: Supplementary file 1 [file Table_1.pdf]

Supplementary Table S1 The diagnostic criteria of cCR and near-cCR of our study

Diagnostic criteria	cCR	near-cCR
Endoscopy	Flat, white scar<br>Telangiectasia<br>No ulcer<br>No nodularity<br>Negative of multiple biopsies	Irregular mucosa<br>Small mucosal nodules or minor mucosal abnormality<br>Superficial ulceration<br>Mild persisting erythema of the scar<br>Negative of multiple biopsies
Digital Rectal Exam	Normal	Smooth induration or minor mucosal abnormalities
MRI of the pelvic-T2W	Only dark T2 signal, no intermediate T2 signal; and no visible lymph node	Mostly dark T2 signal, some remaining intermediate signal; and/or partial regression of lymph nodes
MRI of the pelvic-DW	No visible tumor on B800-B1000 signal； and/or lack of signal on ADC map； Uniform, linear signal in wall above tumor is ok	Significant regression of signal on B800-B1000； and/or minimal or low residual signal on ADC map
Transrectal ultrasound	No visible tumor； and no visible lymph node	Significant tumor regression, with only minimal remaining bowel wall thickening or small lymph nodes (largest short diameter less than 8 mm)
Serum CEA	Normal	Normal
Enhanced CT of chest and abdomen	No distant metastasis	No distant metastasis

Abbreviation: ADC, apparent diffusion coefficient; cCR, clinical complete response; CEA, carcinoembryonic antigen; DW, diffusion weighted; MRI, magnetic resonance imaging; near-cCR, near clinical complete response; T2W, T2-weighted.
